# Ranking of physiotherapeutic evaluation methods as outcome measures of stifle functionality in dogs

**DOI:** 10.1186/1751-0147-55-29

**Published:** 2013-04-08

**Authors:** Heli K Hyytiäinen, Sari H Mölsä, Jouni T Junnila, Outi M Laitinen-Vapaavuori, Anna K Hielm-Björkman

**Affiliations:** 1 Department of Equine and Small Animal Medicine, Faculty of Veterinary Medicine, University of Helsinki, Viikintie 49, P.O. Box 57 , Helsinki, 00014, Finland; 2 Oy 4Pharma Ltd., Ahventie 4, Espoo, 02170, Finland

**Keywords:** Canine, Stifle, Cranial cruciate ligament, Osteoarthrosis, Physiotherapy, Evaluation, Functional, Testing

## Abstract

**Background:**

Various physiotherapeutic evaluation methods are used to assess the functionality of dogs with stifle problems. Neither validity nor sensitivity of these methods has been investigated. This study aimed to determine the most valid and sensitive physiotherapeutic evaluation methods for assessing functional capacity in hind limbs of dogs with stifle problems and to serve as a basis for developing an indexed test for these dogs. A group of 43 dogs with unilateral surgically treated cranial cruciate ligament deficiency and osteoarthritic findings was used to test different physiotherapeutic evaluation methods. Twenty-one healthy dogs served as the control group and were used to determine normal variation in static weight bearing and range of motion.

The protocol consisted of 14 different evaluation methods: visual evaluation of lameness, visual evaluation of diagonal movement, visual evaluation of functional active range of motion and difference in thrust of hind limbs via functional tests (sit-to-move and lie-to-move), movement in stairs, evaluation of hind limb muscle atrophy, manual evaluation of hind limb static weight bearing, quantitative measurement of static weight bearing of hind limbs with bathroom scales, and passive range of motion of hind limb stifle (flexion and extension) and tarsal (flexion and extension) joints using a universal goniometer. The results were compared with those from an orthopaedic examination, force plate analysis, radiographic evaluation, and a conclusive assessment. Congruity of the methods was assessed with a combination of three statistical approaches (Fisher’s exact test and two differently calculated proportions of agreeing observations), and the components were ranked from best to worst. Sensitivities of all of the physiotherapeutic evaluation methods against each standard were calculated.

**Results:**

Evaluation of asymmetry in a sitting and lying position, assessment of muscle atrophy, manual and measured static weight bearing, and measurement of stifle passive range of motion were the most valid and sensitive physiotherapeutic evaluation methods.

**Conclusions:**

Ranking of the various physiotherapeutic evaluation methods was accomplished. Several of these methods can be considered valid and sensitive when examining the functionality of dogs with stifle problems.

## Background

The role of animal physiotherapy has grown tremendously in modern post-operative rehabilitation of canine orthopaedic patients. The tests used to evaluate patients are mostly adapted from human physiotherapy. Although the assessment protocols may have been tested for reliability and validity in humans, this is not the case in dogs.

Cranial cruciate ligament deficiency (CCLd) is one of the most common causes for hind limb lameness in dogs [1-3]. Numerous surgical treatment methods exist, and post-operative rehabilitation by animal physiotherapists is part of a successful outcome [4]. Osteoarthrosis (OA) is known to follow CCLd, regardless of the chosen surgical treatment method [1,5]. Several reports of the effectiveness of physiotherapeutic intervention for dogs with stifle problems already exist [3,4,6]. However, the evaluation of success of physiotherapeutic treatment is subjective, since validated outcome assessment methods are lacking in veterinary medicine.

Various methods to evaluate the functional status of the dogs with surgically treated (ST) CCLd have been used by physiotherapists [7-9]. The results are often described in a qualitative and subjective manner. To gain reliability for the evaluation, the validity and sensitivity of the methods should be tested.

The aim of this study was to determine the most valid and sensitive physiotherapeutic evaluation methods for assessing functionality of hind limbs in dogs with stifle problems.

## Methods

Two groups of dogs were evaluated. The study group consisted of 43 dogs, 19 males and 24 females, with unilateral ST CCLd that had been surgically treated at least one year earlier. Their average age (±SD) was 7.0 ±2.5 years and body weight 37.6 ±9.4 kg. This group comprised 15 Labrador Retrievers, 6 Rottweilers, 3 Golden Retrievers, 3 mixed breed dogs, 2 Bernese Mountain Dogs, 2 Newfoundland Dogs, 2 Nova Scotia Duck Tolling Retrievers, and 1 of each of the following: Black Russian Terrier, Bordeaux Dog, Bullmastiff, Collie, Dalmatian Dog, Doberman Pincher, Giant Schnauzer, Karelian Bear Dog, Pointer, and Short-Haired German Pointer.

The control group consisted of 21 dogs, 7 males and 14 females, with intact CCLs and no diagnosed musculoskeletal problems. Their average age (±SD) was 3.2 ±1.6 years and body weight 35.5 ±8.3 kg. These dogs also had radiographic screening results that were free of hip dysplasia according to the Federation Cynologique Internationale screening protocol (grade A or B). The breeds comprised 12 Labrador Retrievers and 9 Rottweilers.

The study design was prospective. It was approved by the University of Helsinki Ethics Review Board (UHERB) at Viikki Campus, and a written consent from dog owners was obtained.

### Order of physiotherapeutic evaluation protocol

The physiotherapeutic evaluation was limited to 20 min and included 14 different evaluation methods. All physiotherapeutic evaluation methods are described in Table 1. The tests are numbered (here given in parentheses) for the reader’s convenience. The evaluation was always done in the following order to avoid bias: visual evaluation of lameness (1), visual evaluation of diagonal movement (2), visual evaluation of functional active range of motion (AROM); sitting (3) and lying (5) and difference in thrust of hind limbs through functional tests; sit-to-move (4) and lie-to-move (6), movement in stairs (7), evaluation of hind limb muscle atrophy (8), manual evaluation of hind limb static weight bearing (meSWB) (9), quantitative measurement of static weight bearing (qmSWB) of hind limbs with bathroom scales (10), and passive range of motion (PROM) of hind limb stifle flexion (11) and extension (12), and tarsal flexion (13) and extension (14) of joints, using a universal goniometer (UG).

**Table 1:** Physiotherapeutic evaluation protocol

	
1. Visual evaluation of lameness	The evaluation was performed outside, on a non-slip pavement surface, with the dog on a leash trotting 50 m in a straight line, and movements were observed twice from the front, back and on both sides of the dog.
	The handlers were instructed to move at a brisk walking speed, with the dog moving in a relaxed trot speed at either side of the handler; heel-command was not recommended. If any oblique movement was noted, the handler was asked to present the dog both on their right and left to rule out any effect of excess eye contact between the dog and the handler.
	Movements were also observed in circles, 2–3 m in diameter, leading the dog in both directions.
2. Visual evaluation of diagonal movement	Possible oblique body position during movement in straight lines was noted: moving diagonally in three lines, hindquarters to the right or left.
3.-6. Visual evaluation of functional active range of motion (AROM) (3.-4.) and thrust of hind limbs (5.-6.)	*“Sit (3.)* and *sit-to-move (4.)”* The dog was led over a 20-m distance and asked to sit and sit-to-move 3 times within equal distances. Any functional limitation or compensation of the sitting position, such as external rotation, abduction, limited flexion of the hind limbs, was noted. Observed weakness or asymmetry in thrust of hind limbs from the ground was noted.
	“*Lie down (5.)* and *lie-to-move (6.)”* were done using a similar protocol.
7. Movement in stairs	The stairs used for testing were 15 cm high, 30 cm deep, and 2 m wide indoor stairs with solid steps and un-slippery surface. The dog was led and the handler was instructed to perform a controlled climb up and down the stairs. This was done twice and observed from both above and below in turn.
8. Manual evaluation of hind limb muscle atrophy	The dog stood in a symmetrical, square position, with the owner holding the dog straight. The symmetry of the width of the muscle bulk of both hind limbs was evaluated manually by palpating and comparing them simultaneously. This was done for both the cranial and caudal thigh muscle groups.
9. Manual evaluation of static weight bearing of hind limbs (meSWB)	Static weight bearing of the hind limbs was evaluated manually by lifting each of the limbs in turn and evaluating possible differences in resistance; a weaker resistance was noted.
10. Quantitative measurement of static weight bearing of hind limbs (qmSWB)	SWB was also measured with the hind limbs placed on two identical digital scales (Medica plus M-135, Truebell Vantaa, Finland). The scales had a measurement accuracy of 0.1 kg and a measurement range from 3 kg to 150 kg. The scales gave a stationary final score. The measurement protocol is described elsewhere [10]. Measurements were recorded in kilograms, with an accuracy of two decimals, and the mean values for each dog were calculated.
11.-14. Measurement of passive range of motion: PROM of stifle (11.-12.) and tarsal joints (13.-14.)	The PROM of stifle and tarsal joints was measured from unsedated dogs using a small-sized, flexible 180° UG with a 5° scale. The dog was placed in lateral recumbency, where the handler maintained the position of the dog, and the examiner was situated caudally to the dog. Joints proximal to the one being measured were positioned so that the least amount of muscular restriction affected the joint measured.
	Measurement procedure followed standard joint measurement protocols, where the UG was placed lateral to the joint in question, and the axis of the UG was placed over the axis of the movement of the joint. The stationary arm of the UG lied parallel to the longitudinal axis of the bone proximal to the joint and pointed towards the greater trochanter of the femur when the PROM of the stifle joint was measured, and towards the extensor groove and the tibial tuberosity when the tarsal joint was measured.
	The movable arm of the UG lied parallel to the longitudinal axis of the bone distal to the joint segment and pointed towards the lateral malleolus of the fibula when the PROM of the stifle joint was measured, and towards the distal end of the fifth metatarsus when the tarsal joint was measured.
	Three measurements of each joint in maximal flexion and extension from both hind limbs were taken. All of the same-side measurements were taken alternately between the two joints before the dog’s side was changed. The PROM in extension and flexion was followed through until the last possible end of PROM was met at the furthest possible full fifth degree, limited by either active resistance of the dog, pain, or palpable end-feel. Possible deviant findings in end-feels and limiting factors were recorded.

For each task, the handler of the dog, usually the owner, was given standardized instructions. The tests were always performed in the same environment. In case of disturbance (e.g. reaction to other dogs, misbehaviour), the handler was asked to repeat the tasks more often than mentioned in the protocol. Assistive aids, such as treats or toys, were used to motivate the dogs to perform tasks, if needed. An assistant recorded the results.

A numerical evaluation was used to describe the dog’s level of performance in each task as follows:

Visual evaluation of lameness, diagonal movement and movement in stairs

Tasks are described as physiotherapy tests 1,2, and 7 in Table 1. Movement was graded as 0 = normal movement and equal weight bearing, 1 = random asymmetry of movement, 2 = obvious asymmetry of movement (e.g. abnormal movement patterns such as abduction during swing phase, decrease or increase in either caudal or cranial stance phase, bunny-hopping or weight bearing only in one direction in stairs), and 3 = weight bearing or non-weight bearing lameness, in stairs constant misstepping.

In addition, possible oblique body position during movement was noted: 1 = symmetrical, 2 = oblique, hindquarters to the right, 3 = oblique, hindquarters to the left.

Visual evaluation of functional AROM and thrust of hind limbs

Tasks are described as physiotherapy tests 3–6 in Table 1. Active range of motion during sitting and lying position was graded based on the visually evaluated position of the hind limb. Possible external rotation, decrease in flexion of stifle and tarsus, and abduction of the limb were evaluated as follows: 1 = no findings, 2 = any finding in left limb, 3 = any finding in right limb, 4 = bilateral finding in hind limbs.

Symmetry in hind limb thrust from sitting and lying positions to standing position was classified as 1 = symmetrical thrust between hind limbs, 2 = less thrust in left hind limb, or 3 = less thrust in right hind limb.

Atrophy

Task is described as physiotherapy test 8 in Table 1. The hind limbs were assessed to have either 1 = symmetrical muscle mass in hind limbs, 2 = decreased muscle mass in left hind limb, or 3 = decreased muscle mass in right hind limb.

Manual evaluation and quantitative measurement of static weight bearing of hind limbs and measurement of PROM of stifle and tarsal joints

Tasks are described as physiotherapy tests 9–14 in Table 1. In meSWB, the dog was evaluated to have weight bearing, that was either 1 = symmetrical, 2 = decreased on the left hind limb, or 3 = decreased on the right hind limb. The normal limits for qmSWB and measurement of PROM were constructed from the results of the control dogs. Mean percentage difference between control dogs’ hind limbs was used as the normal limit for qmSWB [10]. Similarly, mean + SD and mean - SD were used as the normal limit for flexion and extension variables, respectively. In conclusion, normal values for qmSWB were < 6.05%, for stifle and tarsus flexion < 51.7° and < 40.1°, respectively, and for stifle and tarsus extension > 147.7° and > 169.5°, respectively. Based on these values, the dogs were classified as either 1 = symmetrical qmSWB, 2 = decreased qmSWB in the left hind limb, 3 = decreased qmSWB in the right hind limb. PROM was graded as 1 = bilaterally normal PROM, 2 = decreased PROM in the left hind limb, 3 = decreased PROM in the right hind limb, or 4 = bilaterally decreased PROM.

### Orthopaedic examination

An orthopaedic examination consisting of palpation of the limbs and spine, evaluation of conscious proprioception and withdrawal reflex, and lameness evaluation through a five-point grading [5] was done by an experienced surgeon. Based on the examination, dogs were grouped as follows 1 = no findings in hind limbs, findings in either 2 = left or 3 = right hind limb, or 4 = bilaterally abnormal.

### Force platform analysis

Signals from a force platform (Kistler Type 9286, Kistler Instrumente AG Winterhur, CH-8408, Switzerland) and a start-interrupt timer system were processed with a computer-based software program (Aquire 7.3, Sharon Software Inc., DeWitt, MI, USA). The setup has been described elsewhere [11]. Five valid runs over the force plate at a velocity of 1.70-2.50 m/s and acceleration of -0.5 to +0.5 m/s^2^ were recorded. Means of body weight-corrected peak vertical force (PVF) and impulse (IMP) were calculated. Based on these means ± SD and the difference between left and right limbs by [-|mean difference|-SD ; |mean difference| + SD], dogs were classified as 1 = hind limbs symmetrical or applying less force on either 2 = left or 3 = right hind limb.

### Radiological evaluation

Radiographs of stifle and hip joints were taken under sedation bilaterally from the dogs in the study group. Mediolateral and craniocaudal views were taken from stifle joints. An extended ventrodorsal view was taken from the hip joints. Radiographs were graded according to the amount of OA seen, using a scale from 0 to 3, where 0 = no OA findings, 1 = mild OA findings, 2 = moderate OA findings, and 3 = severe OA findings [12]. Based on these, the dogs were classified as 1 = bilaterally no signs of OA in either stifle or hip joint, or having a radiological stifle or hip OA in either 2 = left or 3 = right stifle or hip joint, or 4 = bilaterally.

### Conclusive assessment

Finally, a conclusive assessment of the hind limbs was done by combining the results of the orthopaedic, force platform, and radiographic evaluations. The hind limbs were classified as 1 = no findings in hind limbs, findings in either 2 = left, 3 = right hind limb, or 4 = bilaterally.

All physiotherapeutic evaluations were performed by the same qualified physiotherapist specialized in veterinary physiotherapy (HH). The orthopaedic examination, force platform study, and conclusive assessment were also performed by one examiner (SM). Radiographs were evaluated by two examiners (AH-B, OL-V). During orthopaedic examination and physiotherapeutic evaluations the examiners were blinded to the surgically treated limb, at least until palpation of possible scar tissue.

### Testing and comparison of the tasks within the protocol

All 14 physiotherapeutic evaluation methods were compared with the results of the 6 veterinarian-used evaluation methods: orthopaedic examination, force platform analysis including PVF and IMP, radiological evaluation of only stifles, and stifles and hips together, and conclusive assessment (hereafter these will be referred to as “the standards”). This was done to determine how well each evaluation method was able to recognize the functionally symptomatic hind limb.

### Statistical methods

Congruity between the 14 different physiotherapeutic evaluation methods and the six standards was evaluated with three different statistical approaches. Three methods were selected because none alone could answer the question of congruity fully. Together, however, they are able to provide a plausible assessment of the reliability of the methods, as the weaknesses of these methods partly cancel each other out.

Fisher’s exact test was first used to evaluate the significance of the association between each physiotherapeutic evaluation method and each standard. Secondly, the proportion of observations where the evaluation method and standard agreed was calculated. Thirdly, similar proportions were calculated, but here agreement was also granted for observations where the evaluation method at hand resulted in an “asymptomatic” and the standard resulted in a “symptomatic” finding. This was done to account for the difference in assessing some variables into four groups and others only into three, as some methods cannot differentiate bilaterally symptomatic from bilaterally asymptomatic. This is referred to as the adjusted proportion of agreement.

The 14 physiotherapeutic evaluation methods were then ranked based on each of these three statistical approaches within the six standards. Thus, 18 different ranking lists were constructed, one for each standard – statistical approach combination. The three rankings within a standard were then summed to place the evaluation methods into a total rank order within a standard. Finally, these six ranking numbers were summed over the standards. A final ranking list was then constructed based on these sums, where the first evaluation method of the list was considered to be the most congruent one (i.e. smallest ranking over all methods and standards combined). Further, sensitivities of all of the physiotherapeutic evaluation methods were calculated against each of the six standards. Statistical analyses were done using SAS® System for Windows, version 9.3 (SAS Institute Inc., Cary, NC, USA).

## Results

The difference between ages of the two groups was highly significant (*P* < 0.0001). Three dogs in the study group either did not have platform analysis or radiographs taken due to uncooperativeness or poor general condition of the dog. Due to behavioural problems, two dogs did not tolerate manual evaluation of weight bearing. Out of the tested dogs, two performed the runs over the force plate in range of 1.72-2.10 m/s, and all of the rest within range of 2.10–2.50 m/s.

The comparison of various physiotherapeutic evaluation methods with the standards is presented in Table 2. Significant associations between methods are indicated with an asterisk.

**Table 2:** Association between physiotherapeutic evaluation methods and standards based on three statistical approaches

	**Peak vertical force**			**Vertical impulse**			**Orthopaedic examination**			**Conclusive assessment**			**Stifle radiographs**			**Stifle + hip radiographs**		
	**FET**	**PA**	**APA**	**FET**	**PA**	**APA**	**FET**	**PA**	**APA**	**FET**	**PA**	**APA**	**FET**	**PA**	**APA**	**FET**	**PA**	**APA**
	***P-*value**	**(%)**	**(%)**	***P-*value**	**(%)**	**(%)**	***P-*value**	**(%)**	**(%)**	***P-*value**	**(%)**	**(%)**	***P-*value**	**(%)**	**(%)**	***P-*value**	**(%)**	**(%)**
1. Visual evaluation of lameness	0.607	43	67.5	0.559	48	67.5	0.865	21	86	0.586	15	85	0.92	19	83.3	0.864	26	81
2. Diagonal movement	0.142	33	75	0.378	45	77.5	0.761	21	90.7	0.598	18	90	0.664	21	90.5	1	19	88.1
3. Sitting position	0.858	41	56.8	0.249	35	54.1	0.003*	43	97.5	<.001*	51	100	0.001*	49	94.9	<001*	56	94.9
4. Thrust from sitting	0.386	38	56.8	0.273	41	56.8	0.154	58	92.5	0.041*	60	94.6	0.025*	62	92.3	0.009*	59	89.7
5. Lying positon	0.898	38	64.9	0.322	38	62.2	0.013*	33	100	0.007*	38	100	0.006*	36	94.9	0.011*	36	94.9
6. Thrust from lying	0.407	38	59.5	0.897	41	56.8	0.107	55	92.5	0.032*	60	97.3	0.025*	54	92.3	0.032*	51	89.7
7. Stairs	0.042*	55	81.6	0.031*	55	76.3	0.286	15	89.7	0.39	18	92.1	0.19	16	86.8	0.175	21	86.8
8. Evaluation of atrophy	0.018*	42	52.6	0.004*	47	53.6	0.001*	78	97.6	<.001*	79	97.4	0.001*	75	94.9	0.001*	73	90
9. meSWB	0.002*	49	65.7	0.021*	57	62.9	0.207	55	89.5	0.14	57	91.4	0.176	54	86.5	0.15	51	83.8
10. qmSWB	0.082	51	82.1	0.032*	62	82	0.149	37	97.6	0.111	36	97.4	0.411	37	95.1	0.035*	37	95.1
11. Stifle flexion	0.598	46	71.8	0.811	44	66.7	0.052	22	97.6	0.027	23	97.4	0.004*	27	97.6	0.005*	29	97.6
12. Stifle extension	0.499	33	59	0.891	39	56.4	0.004*	34	92.7	0.003*	33	92.3	0.004*	37	95.1	0.001*	42	95.1
13. Tarsus flexion	0.477	21	56.4	0.166	18	51.3	0.028*	51	80.5	0.02*	54	82.1	0.213	39	92.7	0.144	42	92.7
14. Tarsus extension	0.216	36	71.8	0.242	41	71.8	0.308	12	85.4	0.262	13	84.6	0.093	7.3	92.7	0.032*	12	90.2

Significant associations are indicated with an asterisk (*). Abbreviations: manual evaluation of static weight bearing (meSWB), quantitative measurement of static weight bearing (qmSWB). Fisher’s exact Test (FET), Proportion of agreement (PA) and Adjusted proportion of agreement (APA). The physiotherapeutic evaluation methods are numbered as in Table 1. N = 38–43.

The only physiotherapeutic evaluation method that had a significant association with all of the standards was assessment of atrophy. The functional tests of sitting, lying, and thrust from both positions had significant associations with three or four of the standards, as did the measurement of the stifle PROM. Tarsus PROM, meSWB, qmSWB, and stairs had significant associations with only one or two standards. Visual evaluation of lameness and diagonal movement did not have significant associations with any of the standards.

The synopsis of the physiotherapeutic evaluation method ranking shows that the functional tests of sitting, lying, and rising from both positions, evaluation of atrophy, meSWB, and qmSWB, in addition to measurement of PROM of the stifle joint, ranked as the highest (Table 3).

**Table 3:** Physiotherapeutic evaluation methods in rank order and the sensitivity of each method

**Ranking order**	**Test**	**Orthopaedic examination**	**Conclusive assessment**	**Stifle radiographs**	**Stifle/hip radiographs**	**PVF**	**IMP**
1	8. Evaluation of atrophy	1	1	1	3	6	6
		80.50%	81.60%	82.10%	82.10%	80.00%	87.50%
2	3. Sitting positon	2	2	2	2	2	2
		70.00%	67.60%	68.40%	68.40%	68.40%	56.30%
3.	10. qmSWB	6	6	8	7	2	1
		39.00%	38.50%	40.00%	40.00%	40.00%	50.00%
4	11. Stifle flexion	8	7	4	4	4	8
		43.90%	43.60%	42.50%	42.50%	50.00%	43.80%
5	5. Lying position	3	3	6	6	11	10
		47.50%	51.40%	47.40%	47.40%	47.40%	43.80%
6	4. Thrust from sitting	5	5	5	5	10	9
		65.00%	64.90%	68.40%	68.40%	63.20%	62.50%
7	12. Stifle extension	4	8	3	2	13	14
		63.40%	64.10%	62.50%	62.50%	65.00%	68.80%
8	9. meSWB	9	10	10	10	3	3
		65.80%	65.70%	66.70%	66.70%	64.70%	85.70%
9	6. Thrust from lying	7	4	7	8	9	13
		62.50%	62.20%	60.50%	60.50%	57.90%	62.50%
10	7. Stairs	12	11	13	12	7	5
		41.70%	41.70%	41.70%	41.70%	52.60%	50.00%
11	2. Diagonal movement	11	12	12	13	5	4
		30.20%	27.50%	29.30%	29.30%	15.00%	23.50%
13	14. Tarsus extension	14	14	11	11	7	5
		36.60%	35.90%	35.00%	35.00%	40.00%	37.50%
12	13. Tarsus flexion	10	9	9	9	14	12
		43.90%	43.60%	42.50%	42.50%	50.00%	43.80%
14	1. Visual evaluation of lameness	13	13	14	14	8	7
		50.00%	48.70%	51.30%	51.30%	52.60%	56.30%

Rank order is presented in the left column, and the ranking of compared methods is specified for each test in the top row of each ranked test. Sensitivities are presented as percentages; sensitivities did not affect rank order. Abbreviations: manual evaluation of static weight bearing (meSWB), quantitative measurement of static weight bearing (qmSWB), peak vertical force (PVF), and vertical impulse (IMP). The physiotherapeutic evaluation methods are numbered as in Table 1.

Sensitivities of the physiotherapeutic methods are also presented in Table 3. Sensitivity of evaluation of atrophy ranged between 80% and 87.5% when tested against any of the six standards. Sensitivity of sitting position ranged between 67.6% and 70.0%, except when compared with the IMP, where the sensitivity was 56.3%. MeSWB had a sensitivity ranging from 64.7% to 85.7%. Thrust from a lying position had a sensitivity ranging between 60.5% and 62.5% with all other standards, except PVF, where the sensitivity was 57.9%. Stifle extension and thrust from sitting position had sensitivities of 62.5-68.8% and 62.5-68.4%, respectively.

## Discussion

Physiotherapeutic evaluation methods used in this study were chosen based on face validity from the available literature, expert advice, and experience from years of rehabilitating patients with ST CCLd, OA, and other stifle problems. The goal was to find the best possible methods to identify possibly decreased functional performance level of these dogs’ hind limbs.

### Visual evaluation of lameness

Visual evaluation of lameness did not prove to be a reliable method in this study. Despite good intra-tester reliability, earlier studies have also noted a poor correlation with objective data, along with weak interobserver reliability and accuracy [13-16].

Lameness evaluation was performed in both the orthopaedic examination by the veterinarian and as part of the physiotherapeutic evaluation by the veterinary physiotherapist. A physiotherapist is not only interested in the degree of lameness but also in its quality. An example would be decreased retraction, lower arch of swing phase in protraction combined with abduction and medial rotation. This might implicate weakness in the biceps femoris and tightness in the gluteus medius [17]. Previous literature has shown that verbal rating scales and numerical rating scales should not be compared [16]. In our study, the visual lameness scoring used by the veterinary surgeon was numerical, and the one used by the physiotherapist was verbal, and thus, they were not comparable.

Diagonal movement has been considered to be informative as to whether or not the dog is compensating the use of one of the hind limbs [18]. It has been assumed that if a dog moves diagonally on three lines, e.g. hind quarters to the right, the compensation would be to alter weight bearing between hind limbs so that the right hind limb carries less weight during stance phase than the left, which is brought to the midline. This would indicate a problem in the right hind limb. However, in the present study, diagonal movement did not correlate with any other evaluation method and was ranked in the lowest third of all methods evaluated. It should therefore no longer be used as an indicator of problematic stifle.

### Functional tests

Functionality in this context refers to the dog’s ability to perform tasks related to activities of daily living and is the main factor influencing the dogs quality of life. It should therefore be assessed more thoroughly.

Sitting position and rising are considered very informative when evaluating stifle patients [7,8]. Even though functional tasks are commonly used for evaluation in the field of human physiotherapy [19-21], they have not been thoroughly tested in veterinary medicine. In this study, the basic functional tasks of sitting and lying down and asymmetry of thrust in hind limbs whilst rising from these positions were assessed. These methods of evaluation were all valid and sensitive in detecting stifle problems, supporting the use of these functional tasks in the evaluation of treatment outcome.

### Muscle mass: manual evaluation of symmetry

The subjective assessment of muscle mass symmetry of the standing dog is a typical evaluation method in a clinical setting [18]. The measurement of thigh circumference using a tape measure has been reported in several studies [7,9,22,23]. Our results show that even the subjective evaluation of asymmetry in thigh muscle mass is a sensitive method for detecting functional problems. Nevertheless, the test could be improved by measuring atrophy with a tape measure, thus quantifying the measurement and increasing the objectivity of the method. Reliability of different types of tape measures in measuring thigh circumference of dogs has been reported to be high, i.e. good repeatability within the measurement device, and greater intra- than interobserver variation has been noted [24]. Nevertheless, clinical experience has shown that obtaining reliable results with a tape measure can be challenging in the case of furry animals, angulations in hind limb structure, and great variation in the shape and length of the femur between different breeds and in the positioning and size of the tape measure.

### Static weight bearing

Clinical experience indicates that, in dogs with ST CCLd, asymmetry in SWB remains a problem even after asymmetry in dynamic weight bearing has resolved [23]. While manual evaluation of SWB can be fairly accurate, it remains a subjective method. Measuring the SWB with bathroom scales provides an objective, reliable quantitative outcome measure for dogs with OA changes in hind limb joints [10]. In this study, qmSWB and meSWB ranked third and seventh among the physiotherapeutic evaluation methods.

### PROM

The concept of functional ROM has been discussed in a study of dogs with CCL treated surgically with the tibial plateau levelling osteotomy (TPLO) technique [25]. A loss greater than 10° in either flexion or extension was associated with a higher lameness score, whereas a limitation in ROM of less than 10° was not associated with a significant increase in clinical lameness. In our study, the PROM of the stifle was found to be an important measurement. Surprisingly, despite the reciprocal system connecting the movement of the stifle and the tarsus in the dog, the ROM of the tarsus proved not to be equally important; in fact it was one of the weakest methods. This may indicate that the effect of the reciprocal system can be eliminated through careful positioning of joints during PROM measurements, but based on the findings of evaluation of sitting and lying positions, i.e. evaluation of the AROM, the effect of the reciprocal system is larger. Special effort was directed to standardizing the measurement positions in such a way that the movement of the joint measured was as isolated as possible, unaffected by the position of the other joints, and a full range of motion was sought, as reported in earlier studies [25-27].

Consistent with an earlier report [25], our study showed that atrophy might benefit the PROM in the problematic limb. This is due to the bigger muscle mass in the contralateral limb, filling the angle between the femur and tibia and therefore limiting movement, giving a smaller flexion value. Less muscle mass allows the stifle joint to flex more if no other restrictive factors, such as pain or possibly large osteophyte formation, are present.

The effect of age on the PROM of dogs stifles is currently unknown. In the present study, there was a highly significant difference between ages of the two groups. For the purposes of this study, it was assumed that there are no changes in PROM due to ageing alone, but further research in this area is warranted.

The authors recognize some limitations to the study. Different number of categories in the different evaluation methods is one limitation of the study. Diagonal movement, stairs, thrust from sitting and lying positions, evaluation of atrophy, qmSWB, meSWB, and force platform analysis had only three categories (1 = symmetrical, 2 = left hind limb problem, 3 = right hind limb problem), whereas visual lameness evaluation, evaluation of sitting and lying positions, measurement of PROM, orthopaedic examination, conclusive assessment, and radiological examinations had four categories(1 = bilaterally problem-free, 2 = left hind limb problem, 3 = right hind limb problem, 4 = bilaterally problematic).

When the two types of classification groups were compared, some of the first groups’ symmetrical dogs may have belonged to either the “bilaterally problem-free” or “bilaterally problematic” categories, but the categorization method with three options does not differentiate these two. This was, however, taken into consideration in the statistical analysis by allowing agreement in observations where the result of the four-category method was bilaterally problematic and the result of the three-category method was either a left or a right hind limb problem. Thus, in this case, it was assumed that finding a symptomatic dog was sufficient.

Also, some of the physiotherapeutic evaluation methods aimed at assessing functional status of the dog and the ability of the dog to manage in activities of daily living. The standards may give a result of “symptomatic” regardless of the dog’s actual functional level in its activities of daily living. This was also taken into account in the statistical methods used; in the third statistical approach (adjusted proportion of agreement), when the physiotherapeutic evaluation method resulted in “asymptomatic” and the standard resulted in “symptomatic”, agreement was allowed.

Further, when new variables are validated, there is often a problem that they cannot be compared against similar parameters, and therefore the comparisons have to be done with the best existing ones. Hence we do acknowledge, that the fact that the compared parameters do not measure exactly the same thing, may have an effect to the results to some extent.

## Conclusions

Evaluation of atrophy, sitting position, quantitative measurement of static weight bearing, lying position, stifle flexion and extension, thrust from sitting and lying, and manual evaluation of static weight bearing were the most valid and sensitive in detecting hind limb abnormality in our group of dogs, surgically treated for CCL and suffering from OA. We propose that these methods could be used when evaluating rehabilitation outcome of dogs with stifle problems.

## Abbreviations

AROM: Active range of motion; CCL: Cranial cruciate ligament; IMP: Vertical impulse; meSWB: Manual evaluation of static weight bearing; OA: Osteoarthritis; PROM: Passive range of motion; PVF: Peak vertical force; qmSWB: Quantitative measurement of static weight bearing; ROM: Range of motion; ST CCLd: Surgically treated cranial cruciate ligament deficiency; SWB: Static weight bearing; UG: Universal goniometer

## Competing interests

The authors declare that they have no competing interests.

## Authors’ contributions

HH carried out the physiotherapy evaluation and drafted the manuscript. SM carried out the orthopaedic, radiological, and conclusive evaluations in addition to contributing to the manuscript. JJ performed the statistics and participated in drafting the manuscript. OL-V participated in study design, contributing to the manuscript, and conceiving the study. AH-B participated in drafting the study, study design, and conceiving the study. All authors read and approved the final manuscript.

## References

[B1] Comerford EJ Smith K Hayashi K Update on the aetiopathogenesis of canine cranial cruciate ligament disease Vet Comp Orthop Traumatol 2011 24 91 98 10.3415/VCOT-10-04-0055 21243176

[B2] Jerre S Rehabilitation after extre-articular stabilisation of cranial cruciate ligament rupture in dogs Vet Comp Orthop Traumatol 2007 2 148 152 10.3415/vcot-07-05-0044 19290397

[B3] Marsolais GS Dvorak G Conzemius MG Effects of postoperative rehabilitation on limb function after cranial cruciate ligament repair in dogs J Am Vet Med Ass 2002 9 1325 1330 11991410 10.2460/javma.2002.220.1325

[B4] Monk ML Preston CA McGowan CM Effects of early intensive postoperative physiotherapy on limb function after tibial plateau leveling osteotomy in dogs with deficiency of the cranial cruciate ligament Am J Vet Res 2006 67 529 536 10.2460/ajvr.67.3.529 16506922

[B5] Mostafa AA Griffon DJ Thomas MW Morphometric characteristics of the pelvic limbs of Labrador Retrievers with and without cranial cruciate ligament deficiency Am J Vet Res 2009 70 498 507 10.2460/ajvr.70.4.498 19335106

[B6] Crook T McGowan C Pead M Effect of passive stretching on the range of motion of osteoarthritic joints in 10 Labrador Retrievers Vet Rec 2007 160 545 547 10.1136/vr.160.16.545 17449709

[B7] Canapp SO The canine stifle Clin Tech Small Anim Pract 2007 22 195 205 10.1053/j.ctsap.2007.09.008 18198788

[B8] Hesbach AL Techniques for objective outcome assessment Clin Tech Small Anim Pract 2007 22 146 154 10.1053/j.ctsap.2007.09.002 18198782

[B9] Millis DL Millis DL, Levine D, Taylor RA Assessing and measuring outcomes Canine Rehabilitation & Physical Therapy 2004 1 USA: Saunders 211 227

[B10] Hyytiäinen HK Mölsä SH Junnila JT Laitinen-Vapaavuori OM Hielm-Björkman AK Use of bathroom scales in measuring asymmetry of hindlimb static weight bearing in dogs with osteoarthritis Vet Comp Orthop Traumatol 2012 25 390 396 10.3415/VCOT-11-09-0135 22828919

[B11] Mölsä SH Hielm-Björkman AK Laitinen-Vapaavuori OM Force platform analysis in clinically healthy Rottweilers: comparison with Labrador Retrievers Vet Surg 2010 39 701 707 20345537 10.1111/j.1532-950X.2010.00651.x

[B12] de Rooster H van Bree H Use of compression stress radiography for the detection of partial tears of the canine cruciate ligament J Small Anim Pract 1999 40 573 576 10.1111/j.1748-5827.1999.tb03024.x 10664954

[B13] Fuller CJ Bladon BM Driver AJ Barr ARS The intra- and inter-asessor reliability of measurement of functional outcome by lameness scoring horses Vet J 2006 171 281 286 10.1016/j.tvjl.2004.10.012 16490710

[B14] Quinn MM Keuler NS Lu Y Faria MLE Muir P Markel MD Evaluation of agreement between numerical rating scales, visual analogue scoring scales, and force plate gait analysis in dogs Vet Surg 2007 36 360 367 10.1111/j.1532-950X.2007.00276.x 17547599

[B15] Waxman AS Robinson DA Evans RB Hulse DA Innes JF Conzemius MG Relationship between objective and subjective assessment of limb function in normal dogs with and experimentally induced lameness Vet Surg 2008 37 241 246 10.1111/j.1532-950X.2008.00372.x 18394070

[B16] Hewetson M Christley RM Hunt ID Voute LC Investigations of the reliability of observational gait analysis for the assessment of lameness in horses Vet Rec 2006 24 852 858 16798953 10.1136/vr.158.25.852

[B17] Evans HE de LaHunta A Quide to the Dissection of the Dog 2010 7 Saunders, Elsevier: St. Louis, Missouri 51 58

[B18] Millis DL Taylor AR Hoelzler M Millis DL, Levine D, Taylor RA Orthopedic and neurologic evaluation Canine Rehabilitation & Physical Therapy 2004 1 USA: Saunders 179 210

[B19] Risberg MA Ekeland A Assessment of functional tests after anterior cruciate ligament surgery J Orthop Sports Phys Ther 1994 19 212 217 8173569 10.2519/jospt.1994.19.4.212

[B20] Yilmaz G Baltaci G Evaluation of knee strength, functional performance and sports activity level 18–24 months after anterior cruciate ligament reconstruction Phys Ther Sport 2006 7 178 179 10.1016/j.ptsp.2006.09.015

[B21] Cole B Finch E Gowland C Mayo N ed Basmajian J Physical rehabilitation outcome measures 1995 Baltimore, USA: Williams & Wilkins 1 172

[B22] Allen MJ Leone KA Lamonte K Townsend KL Mann KA Cemented total knee replacement in 24 dogs: surgical technique, clinical results, and complications Vet Surg 2009 38 555 567 10.1111/j.1532-950X.2009.00528.x 19573056

[B23] Moeller EM Allen DA Wilson ER Lineberger JA Lehenbauer T Long-term outcomes of thigh circumference, stifle range-of-motion, and lameness after unilateral tibial plateau leveling osteotomy Vet Comp Orthop Traumatol 2010 23 37 42 19997674 10.3415/VCOT-09-04-0043

[B24] Baker SG Roush JK Unis MD Wodiske T Comparison of four commercial devices to measure limb circumference in dogs Vet Comp Orthop Traumatol 2010 6 406 410 20830452 10.3415/VCOT-10-03-0032

[B25] Jandi AS Schulman AJ Incidence of motion loss of the stifle joint in dogs with naturally occurring cranial cruciate ligament rupture surgically treated with tibial plateau levelling osteotomy: longitudinal clinical study of 412 cases Vet Surg 2007 36 114 121 10.1111/j.1532-950X.2006.00226.x 17335418

[B26] Jaeger G Marcellin-Little DJ Levine D Reliability of goniometry in Labrador Retrievers Am J Vet Res 2002 63 979 989 10.2460/ajvr.2002.63.979 12118679

[B27] Nicholson HL Osmotherly PG Smith BA McGowan CM Determinants of passive hip range of motion in adult Greyhounds Aust Vet J 2007 85 217 221 10.1111/j.1751-0813.2007.00145.x 17547633

